# Differences in the autonomic regulation of temperature in the lower lip and tongue during activation of the lingual nerve

**DOI:** 10.1016/j.jphyss.2025.100028

**Published:** 2025-06-04

**Authors:** Syed Taufiqul Islam, Toshiya Sato, Hanako Ohke, Mari Shimatani, Hisayoshi Ishii

**Affiliations:** a Division of Physiology, Department of Oral Biology, School of Dentistry, Health Sciences University of Hokkaido, Ishikari-Tobetsu, Hokkaido, Japan; b Division of Dental Anesthesiology, Department of Human Biology and Pathophysiology, School of Dentistry, Health Sciences University of Hokkaido, Ishikari-Tobetsu, Hokkaido, Japan

**Keywords:** Noncholinergic parasympathetic vasodilation, Trigeminal mediated reflex, Cervical sympathetic nerves, Orofacial temperature

## Abstract

Orofacial temperature influences orofacial functions and is related to hemodynamics mediated by the autonomic nerves. Although the properties of autonomic vasomotor responses differ in orofacial tissues, differences in the autonomic regulation of orofacial temperature are unclear. We examined the differences in blood flow (BF) and temperature (Tm) between the extraoral (lower lip) and intraoral tissues (tongue) of urethane-anesthetized rats. Noncholinergic parasympathetic vasodilation evoked by trigeminal-mediated reflex elicited significant increases in BF and Tm in both tissues, and these increases were larger in the tongue than in the lower lip. Activation of cervical sympathetic nerves significantly decreased BF and Tm in both tissues. These decreases were restored by parasympathetic reflex vasodilation; the effects were larger in the tongue than in the lower lip. Our results suggest that parasympathetic vasodilation is involved in the maintenance of BF and Tm, and that the effects may be greater in intraoral than in extraoral tissues.

## Highlights

• Prominent parasympathetic vasodilation exists in the intraoral tissues.
• The noncholinergic system contributes to orofacial temperature regulation.
• Parasympathetic vasodilation inhibits orofacial temperature decrease.

## Introduction

Two major autonomic vasomotor fibers, parasympathetic vasodilator and sympathetic vasoconstrictor fibers, have been reported in the extraoral (lip, jaw muscles, and salivary glands) [1], [2], [3], [4] and intraoral tissues (tongue, gingiva, and palate) [5], [6], [7]. Parasympathetic vasodilation is evoked by a trigeminal-mediated reflex [1], [2], [8]. On the other hand, sympathetic vasoconstriction is under tonic control from the superior cervical sympathetic trunk [9], [10], [11]. Activation of these fibers rapidly and markedly changes hemodynamics in the orofacial area, suggesting that autonomic mechanisms and their interactions may be important in the maintenance of blood flow and functions in orofacial tissues.

Our previous study showed that parasympathetic vasodilation evoked by trigeminal afferent inputs elicits significant increases in blood flow and local temperature in extraoral tissues such as the lower lip [12]. Furthermore, decreases in blood flow and local temperature in the lower lip (evoked by cervical sympathetic trunk stimulation) were inhibited significantly by lingual nerve stimulation. These results suggest that parasympathetic and sympathetic vasomotor responses and their interactions are involved in the maintenance of hemodynamics and local temperature in the orofacial area.

Local temperature in the orofacial area is generally considered important for maintenance of orofacial functions, such as oral sensation [13], [14] and wound healing [15], and abnormalities in local temperature may be related to orofacial dysfunction [16], [17], [18]. Although autonomic vasomotor fibers are abundant in intraoral tissues, such as the tongue [19], it remains questionable whether autonomic vasomotor responses are also involved in the regulation of local temperature in the intraoral tissues and whether there are site-specific differences in the regulatory mechanism of hemodynamics and local temperature between extraoral and intraoral areas.

Thus, the present study was designed to examine (1) the effects of parasympathetic vasodilation (evoked by the trigeminal afferent inputs) and sympathetic vasoconstriction (induced by the cervical sympathetic trunk) and (2) their interactions on blood flow and local temperature in the extraoral (lower lip) and intraoral tissues (tongue) in deeply urethane-anesthetized, artificially ventilated, vagotomized, and sympathectomized rats.

In all experiments, the cervical vagus nerve and superior cervical sympathetic trunk were transected bilaterally in the neck before stimulation, which ensured that only nonvagal parasympathetic effects were examined during lingual nerve stimulation in the present study.

## Materials and methods

### Preparation of animals

The experiments were performed on 45 adult male Wistar rats (9–15 weeks of age, weighing 310–480 g). After induction of anesthesia with isoflurane, urethane (1 g/kg) in a volume of 1 ml/100 g body weight was injected subcutaneously into the backs of the animals. Room temperature was maintained at 25 ± 1 °C during the experiments. One femoral vein was cannulated to allow for drug injection, and one femoral artery was cannulated and connected to a Statham pressure transducer to monitor systemic arterial blood pressure (SABP) and heart rate (HR).

The anesthetized animals were intubated, paralyzed by intravenous (iv) injection of pancuronium bromide (Mioblock; Organon, Teknika, Netherlands; 0.6 mg/kg initially, supplemented with 0.4 mg/kg every hour or so after testing the level of anesthesia; see below), and artificially ventilated via a tracheal cannula with a mixture of 50 % air and 50 % O_2_. The ventilator (model SN-480–7; Shinano, Tokyo, Japan) was set to deliver a tidal volume of 8.5–10 cm^3^/kg at a rate of 20–23 breaths/min, and the end-tidal concentration of CO_2_ was determined using an infrared analyzer (Capnomac Ultima; Datex, Helsinki, Finland), as reported elsewhere [7], [12], [20]. Continuous ventilation in this manner has been shown to maintain an end-tidal concentration of CO_2_ at 40–45 mmHg.

The changes in end-tidal CO_2_ following each treatment (from 45 to 35 mmHg) were independent of the changes in blood flow and local temperature measured by the present method (data not shown). Rectal temperature was maintained at 37–38 °C with a heating pad. Before the injection of additional pancuronium bromide, the depth of anesthesia was determined to be adequate by the absence of flexion response to a noxious stimulus, such as pinching the digit for approximately 2 s. The criterion for the maintenance of an adequate depth of anesthesia following paralysis was defined as the absence of reflex elevation of SABP in response to noxious stimulus. When the depth of anesthesia was considered inadequate, additional urethane (intermittent doses of 100 mg/kg, iv) was administered.

At the end of the experiment, all the rats were sacrificed using an overdose (approximately 100 mg, iv) of pentobarbital sodium. Experimental protocols were reviewed and approved by the Animal Ethics and Research Committee and were conducted in accordance with the Regulations for the Care and Use of Laboratory Animals of the Health Sciences University of Hokkaido (No. 24–038). All the animals were cared for in accordance with the recommendations in the current National Research Council guide (Guide for the Care and Use of Laboratory Animals) [21].

### Measurement of cardiovascular parameters and local temperature in the orofacial tissues

**Fig. 1:**
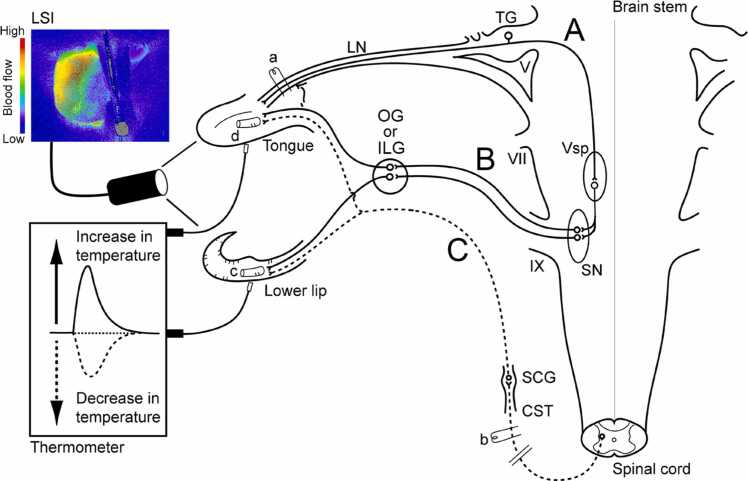
Schematic representation of the electrical stimulation and measurement sites of both blood flow and local temperature in rats. Stimulation sites: peripheral portion of the lingual nerve (LN) (**a**), and peripheral cut end of superior cervical sympathetic trunk (CST) (**b**). Blood flow and local temperature measurement sites: (**c**) lower lip and (**d**) tongue, using laser speckle imaging (LSI) and a thermometer, respectively. The continuous lines indicate: (**A**) trigeminal sensory inputs to the trigeminal spinal nucleus (Vsp) in the brainstem and (**B**) parasympathetic vasodilator fibers to the lower lip and tongue from the salivatory nuclei (SN). The dashed lines indicate: sympathetic vasoconstrictor fibers to the lower lip and tongue from the superior cervical ganglion (SCG) of the superior cervical sympathetic trunk (**C**). Other abbreviations: ILG, intralingual ganglion; OG, otic ganglion; TG, trigeminal ganglion; V, trigeminal nerve root; VII, facial nerve root; IX, glossopharyngeal nerve root.

Modified from Ishii et al. [1].

Changes in blood flow of the lower lip (Fig. 1, c) and tongue (Fig. 1, d) outside the oral cavity were monitored using a laser speckle flowmeter (Omegazone; Omegawave, Tokyo, Japan), which obtains high-resolution 2D blood flow images in seconds, as described previously [4], [7], [12], [22], [23], [24]. A 780-nm semiconductor laser was used to illuminate the surface of the orofacial area. The scattered light was filtered and detected using a CCD camera positioned above the measuring sites. Raw speckle images (real images) corresponding to the number and velocity of moving red blood cells (blood flow) were collected by the CCD camera and transferred to a computer for analysis.

Color-coded blood flow images (speckle images) were obtained in high-resolution mode (638 pixels × 480 pixels; 1 image/s). One blood flow image was generated by averaging the numbers obtained from 20 consecutive raw speckle images. Averaged signals in the blood flow of a region of interest (ROI) were obtained using the pallet software installed in the Omegazone imaging system (Omegawave). The analog output of the equipment did not provide absolute values, but showed relative changes in blood flow (a.u., arbitrary units). The SABP was recorded from a femoral catheter via a Statham pressure transducer. The HR, as well as the systolic, diastolic, and mean SABP, were calculated from the SABP signals (n = 6, Table 1). Vascular conductance was calculated using the following equation:

**Table 1:** Heart rate and systemic arterial blood pressure responses associated each condition.

Heart and blood pressure measurements	Baseline	LN stimulation	CST stimulation	CST + LN stimulation
HR (beats/min)	402 ± 12	416 ± 13	421 ± 13	395 ± 8
Systolic SABP (mmHg)	118.1 ± 2.1	158.5 ± 3.6**	130.5 ± 8.1	159.1 ± 15.7*
Diastolic SABP (mmHg)	65.1 ± 1.1	100.1 ± 4.9**	80.9 ± 8.7	110.8 ± 12.3*
Mean SABP (mmHg)	82.7 ± 1.4	119.5 ± 3.9**	97.5 ± 8.5	126.9 ± 13.2*

Values in table are given as the mean ± standard error of the mean (SEM) (*n* = 6); LN, lingual nerve; CST, cervical sympathetic trunk; HR, heart rate; SABP, systemic arterial blood pressure; Significant difference from baseline at *P < 0.05, **P < 0.001.

Vascular conductance (a.u./mmHg) = blood flow (a.u.)/SABP (mmHg).

Local temperature of the lip and tongue was measured using a non-contact thermometer (PT-3S, OPTEX, Shiga, Japan) (Fig. 1), which measures surface temperature of objects by caching the infrared energy emitted by the target objects (2.5 mm diameter). All data were collected online using a LabScribe2 data-acquisition system (iWorx systems, Washington, NH, USA). Changes (∆) in the parameters were assessed by measuring the height of the maximum (+) or minimum (-) values from the baseline in the responses.

### Electrical stimulation of the lingual nerve and superior cervical sympathetic trunk

The peripheral portion of the lingual nerve (LN) (Fig. 1, a) and the peripheral cut end of the superior cervical sympathetic trunk (Fig. 1, b) were stimulated electrically using a bipolar silver electrode attached to an electrical stimulator (model SEN-7103; Nihon Kohden, Tokyo, Japan). The anterior part of the tongue is innervated by the lingual nerve, which has some branches. This suggests that somatosensory stimulation of the tongue could induce the increases in the blood flow and local temperature due to i) antidromic vasodilation mediated via the axon reflex (activation of sensory fibers with collateral branches to the blood vessels of the tongue) and/or ii) a parasympathetic vasodilation evoked by the trigeminal-mediated reflex. Therefore, the peripheral portion of the intact lingual nerve was electrically stimulated without cutting in the present study. We believe that this stimulation mimics processes occurring in physiological forms of vasomotor responses during orofacial functions, such as mastication, swallowing, and speech. For this purpose, the nerves were stimulated unilaterally under a binocular microscope. The lingual nerve was stimulated for 20 s with supramaximal intensity (100 µA) [7], [20], [24] at various frequencies (1–20 Hz) using 2-ms pulse durations either alone or in combination with cervical sympathetic trunk stimulation (n = 6, in each group). The period of lingual nerve stimulation chosen for the present study was 20 s because the parasympathetic vasodilator fibers were rapidly activated with 20 s stimulation through the trigeminal-mediated reflex, as reported previously [1], [4], [7], [12], [24].

On the other hand, electrical stimulation of the cervical sympathetic trunk was delivered for periods of 2 min with supramaximal intensity (100 µA) [7] at various frequencies (0.5–5 Hz) using 2-ms pulse durations. Cervical sympathetic trunk stimulation for 2 min appears to mimic the physiological forms of spontaneous tonic activity in the fibers supplying the orofacial vasculature [9], [10].

### Pharmacological agents

All drugs were dissolved in sterile saline. To examine whether the lingual nerve stimulation-induced blood flow changes were mediated by a parasympathetic reflex mechanism, lingual nerve stimulation was repeated every 10 min before and after intravenous administration of pharmacological blocking agents. Autonomic ganglion cholinergic blockade was performed using hexamethonium bromide (n = 6, 10 mg/kg; Sigma-Aldrich, St. Louis, MO) and muscarinic cholinergic blockade was performed using atropine sulfate (n = 7, 100 μg/kg; Mitsubishi Tanabe, Osaka, Japan).

These drugs were perfused intravenously for 10 min at a flow rate of 0.1 ml/min using a syringe pump (Model ‘22′ Multisyringe; HARVARD, Holliston, MA). The administration of a similar volume of saline alone had no measurable effect on cardiovascular parameters and local temperature (data not shown). The responses evoked by electrical stimulation after the administration of each drug were determined at least for 7 min after injection because changes in blood flow and SABP reached a steady state during this period. The magnitude of the response obtained following the administration of each agent was expressed as a percentage of the control response recorded prior to the administration of each agent.

The dose of hexamethonium chosen for the present study was 10 mg/kg because a similar dose has been shown to produce a marked inhibition of blood flow increases in the orofacial area evoked by activation of parasympathetic vasodilator fibers through the trigeminal- or vagal-mediated reflex [1], [7], [12], [25], [26]. The efficacy of the blockade using atropine was assessed by the absence of a vasodilator response in response to acetylcholine bromide (1 μg/kg, iv; Sigma-Aldrich). Previous studies showed that acetylcholine bromide at 1 μg/kg induces a similar blood flow increase as that evoked by parasympathetic reflex vasodilation in the masseter muscle [24] and gingiva [7].

### Statistical analysis

All numerical data are presented as mean ± standard error of the mean (SEM). Two-sample data were compared using paired or an unpaired Student's *t*-test. Multiple group comparisons were performed by one-way analysis of variance (ANOVA), followed by post hoc test (Bonferroni/Dunn test) or contrast test. Paired Student's *t*-test (rest vs. each nerve stimulation or drug administration) was used for comparisons of cardiovascular data. The changes in blood flow, vascular conductance and local temperature from basal values or control responses for each treatment were compared using ANOVA followed by Bonferroni/Dunn test. The comparisons between lower lip and tongue were analyzed by unpaired Student's *t*-test or a contrast test. Differences were considered significant at P < 0.05. The data were analyzed using a Macintosh computer with StatView 5.0 (SAS Institute Inc., Cary, NC) and Super ANOVA (ABACUS Concepts, Berkeley, CA) software.

## Results

### Effects of electrical stimulation of the peripheral portion of the lingual nerve on hemodynamics and local temperature in the lower lip and tongue, and SABP

**Fig. 2:**
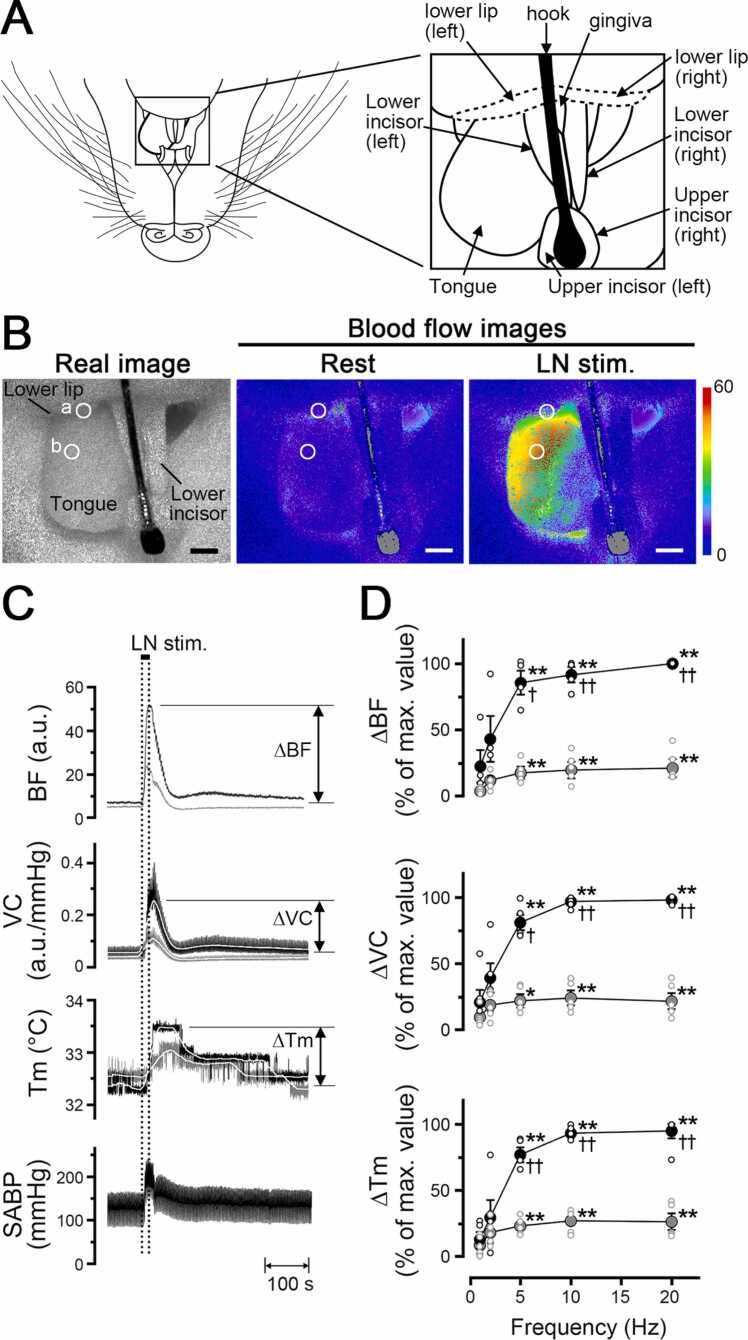
Relationships between hemodynamics and local temperature during trigeminal afferent inputs in the orofacial tissues in the rat. **A**: Illustration of measurement sites of blood flow and temperature in the mandibular area including the lower lip and tongue in a supine rat. **B**: Typical example of the real image and speckle images of the blood flow in the lower lip and inferior surface of the tongue at the basal level (rest) and produced by left lingual nerve stimulation (LN stim.) for 20 s with a supramaximal intensity (100 µA) at 20 Hz using 2-ms pulses. Scale bars represent 3.5 mm. **C**: Increases in blood flow (BF, a.u.; arbitrary units), vascular conductance (VC, a.u./mmHg), and local temperature (Tm, °C) in the lower lip (gray traces) and tongue (black traces) extracted from a region of interest (ROI) indicated by the white circles in **a** and **b**, and systemic arterial blood pressure (SABP, mmHg) evoked by lingual nerve stimulation (horizontal bar with dashed lines). The white traces indicate the mean vascular conductance and local temperature at the measuring sites. **D**: Mean ± standard error of the mean (SEM) of ∆BF, ∆VC, and ∆Tm of the lower lip (gray symbols) and tongue (black symbols) evoked by lingual nerve stimulation at 100 µA and various frequencies (1–20 Hz) (n = 6 in each group). Each value is expressed as a percentage of the maximum response. The statistical significance of the differences from base value (at a frequency of 1 Hz) was assessed by ANOVA followed by a post hoc test (Bonferroni/Dunn test). *P < 0.01, ** P < 0.001 vs. base value. ^†^P < 0.01, ^††^P < 0.001^,^ significant differences between the increases in the lower lip and tongue recorded by lingual nerve stimulation (ANOVA followed by a contrast test). Individual data points are indicated by the white markers.

Fig. 2 shows the changes in the blood flow, vascular conductance, and local temperature in the lower lip and inferior surface of the tongue (Fig. 2A), and the SABP before and after electrical stimulation of the peripheral portion of the lingual nerve. Basal levels for blood flow and vascular conductance in the tongue were significantly higher than those in the lower lip (P < 0.05, paired *t*-test) (Table 2), while the basal levels of local temperature were similar in both sites before lingual nerve stimulation.

**Table 2:** Hemodynamics and local temperature in the orofacial tissues under resting condition.

	Lower lip	Tongue
Blood flow (a.u.)	3.5 ± 0.3	13.7 ± 2.8*
Vascular conductance (a.u./mmHg)	0.05 ± 0.01	0.11 ± 0.02*
Temperature (°C)	33.4 ± 0.6	32.7 ± 0.4

Values in table are given as the mean ± SEM (*n* = 6); Significant difference from lower lip at *P < 0.05.

Electrical stimulation for 20 s with 100 µA at 20 Hz using 2-ms pulses of the left lingual nerve increased blood flow, vascular conductance, and local temperature in both sites on the left side, but not on the right side (Fig. 2B and C). Frequency–response curves were generated using stimulus trains (1–20 Hz) at 100 µA (Fig. 2D). Significant increases in the ∆BF, ∆VC, and ∆Tm evoked by lingual nerve stimulation occurred above 5 Hz (P < 0.001, ANOVA followed by Bonferroni/Dunn test). These increases in the tongue were significantly larger than those in the lower lip when lingual nerve simulation was delivered at 5, 10, and 20 Hz (ANOVA followed by a contrast test).

The results were similar to the responses evoked by lingual nerve stimulation in the dorsum of the tongue (data not shown). The animals exhibited normal systolic and diastolic pressures, mean SABP, and HR during rest (Table 1). The HR remained unchanged during lingual nerve stimulation (paired *t*-test). There were significant differences in the SABP before and after lingual nerve stimulation (P < 0.001) (paired *t*-test).

### Effects of pharmacological blocking agents on hemodynamics and local temperature in the lower lip and tongue evoked by lingual nerve stimulation

**Fig. 3:**
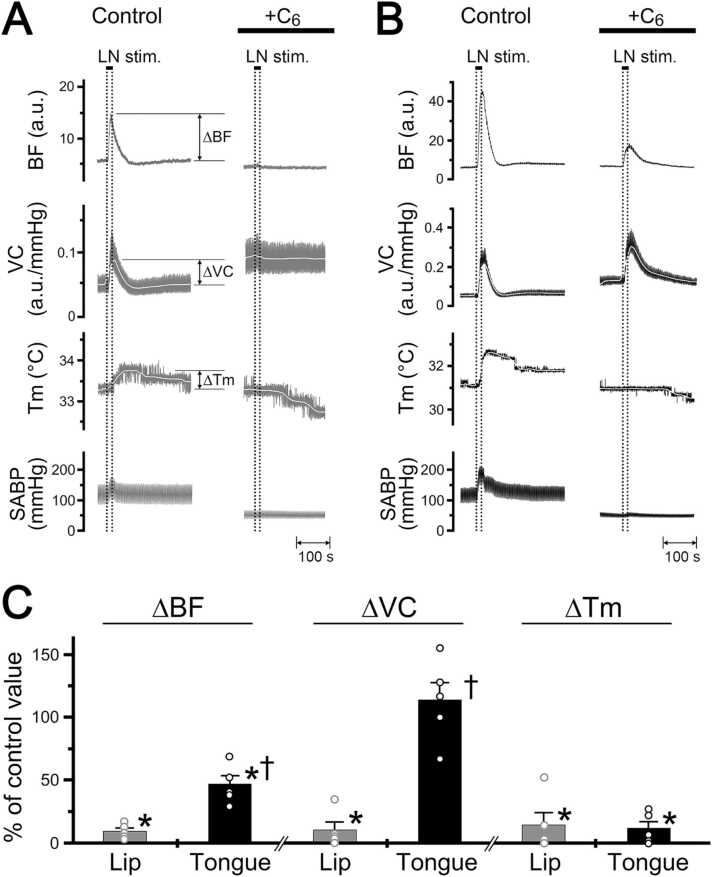
Effects of hexamethonium on changes in the hemodynamics and local temperature in the lower lip and tongue evoked by lingual nerve stimulation. **A**, **B**: Typical examples of the effects of intravenous administration of hexamethonium (C_6_) at 10 mg/kg for 10 min (0.1 ml/min) on changes in blood flow (BF, a.u.; arbitrary units), vascular conductance (VC, a.u./mmHg), and local temperature (Tm, °C) of the lower lip (gray traces, **A**) and tongue (black traces, **B**) on the left side evoked by left lingual nerve stimulation (20 s, 100 µA, 20 Hz, 2 ms). **C**: Mean ± SEM of increases in the ∆BF, ∆VC, and ∆Tm of the lower lip (gray bars) and tongue (black bars) evoked by lingual nerve stimulation with administration of C_6_ (n = 6 in each group). Each value is expressed as a percentage of the control response before treatment. The statistical significance of the differences from the control was assessed by ANOVA followed by a post hoc test (Bonferroni/Dunn test). *P < 0.001 vs. control. ^†^P < 0.01, significant differences between the changes evoked by lingual nerve stimulation in the lower lip and tongue (ANOVA followed by a contrast test). Individual data points are indicated by the white markers.

Fig. 3 shows the effects of intravenous administration of hexamethonium (C_6_, 10 mg/kg) on the increases in blood flow, vascular conductance, and local temperature in the lower lip and tongue evoked by lingual nerve stimulation in rats. Increases in blood flow, vascular conductance, and local temperature in the lower lip on the left side evoked by left lingual nerve stimulation (20 s, 100 µA, 20 Hz, 2 ms) were abolished by the intravenous administration of hexamethonium (10 mg/kg) (Fig. 3A).

There were significant differences in the ∆BF, ∆VC, and ∆Tm in the lower lip before and after hexamethonium administration (P < 0.001, ANOVA followed by Bonferroni/Dunn test) (Fig. 3C). On the other hand, the administration of hexamethonium greatly reduced the increases in blood flow and local temperature in the tongue evoked by lingual nerve stimulation, but it had no effect on the vascular conductance increase in the tongue (Fig. 3B).

There were significant differences in ∆BF and ∆Tm in the tongue before and after hexamethonium administration (P < 0.001), but not in ∆VC (ANOVA followed by Bonferroni/Dunn test) (Fig. 3C). The inhibitory effect of hexamethonium on the increases in blood flow and vascular conductance in the lower lip evoked by lingual nerve stimulation was significantly larger than those in the tongue (ANOVA followed by a contrast test). The response at each site returned to near the initial value at 30–60 min after hexamethonium administration (data not shown).

**Fig. 4:**
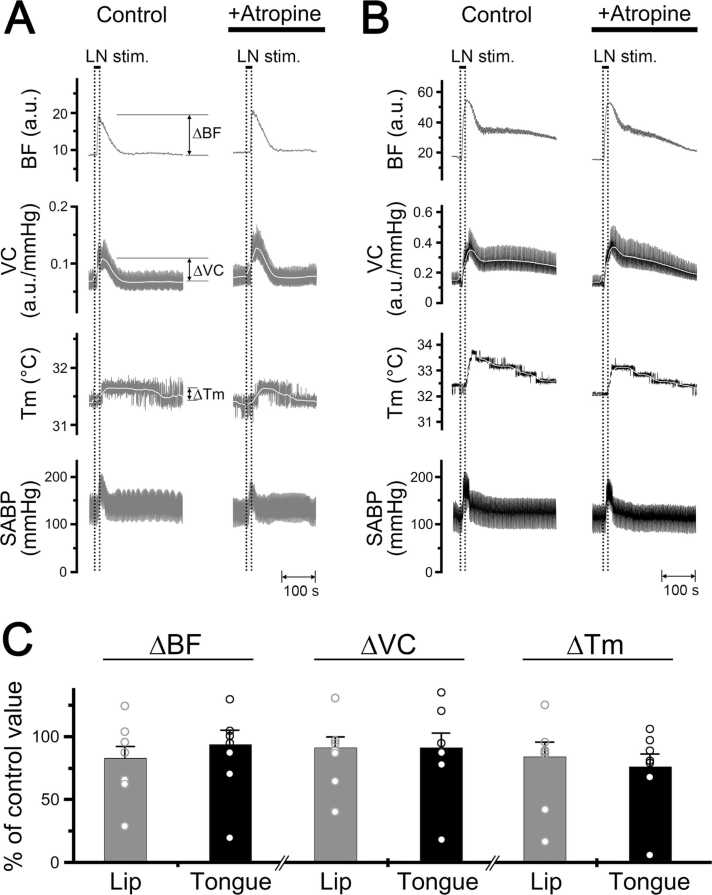
Effects of atropine on changes in the hemodynamics and local temperature in the lower lip and tongue evoked by lingual nerve stimulation. **A**, **B**: Typical examples of the effects of intravenous administration of atropine at 100 μg/kg for 10 min (0.1 ml/min) on changes in blood flow (BF, a.u.; arbitrary units), vascular conductance (VC, a.u./mmHg), and local temperature (Tm, °C) of the lower lip (gray traces, **A**) and tongue (black traces, **B**) on the left side evoked by left lingual nerve stimulation (20 s, 100 µA, 20 Hz, 2 ms). **C**: Mean ± SEM of increases in the ∆BF, ∆VC, and ∆Tm of the lower lip (gray bars) and tongue (black bars) evoked by lingual nerve stimulation with the administration of atropine (n = 7 in each group). Each value is expressed as a percentage of the control response before treatment. The statistical significance of the differences from the control was assessed by ANOVA followed by a post hoc test (Bonferroni/Dunn test). Individual data points are indicated by white markers.

Fig. 4 shows the effects of intravenous administration of atropine (100 μg/kg) on the increases in blood flow, vascular conductance, and local temperature in the lower lip and tongue evoked by lingual nerve stimulation in rats. The administration of atropine had no effect on either site (ANOVA followed by Bonferroni/Dunn test) (Fig. 4). The HR at 10 min after the administration of hexamethonium and atropine was 367 ± 16 and 412 ± 7 beats/min, respectively. The mean SABP after the administration of hexamethonium and atropine was 61.6 ± 2.6 and 103.2 ± 14.1 mmHg, respectively. The HR remained unchanged during lingual nerve stimulation in both treatments (paired *t*-test). There was a statistically significant difference in the mean SABP before and after the administration of hexamethonium (P < 0.001), but not atropine (paired *t*-test).

### Sympathetic effects on hemodynamics and local temperature in the lower lip and tongue, and SABP

**Fig. 5:**
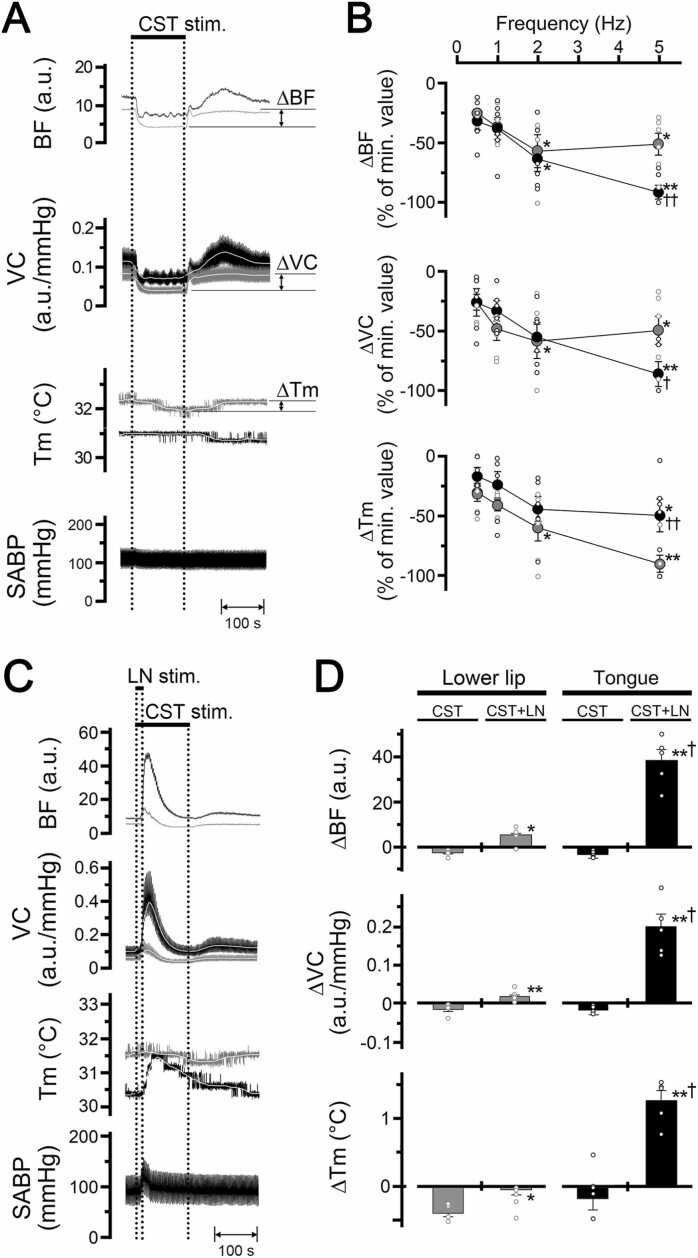
Sympathetic effects on cardiovascular parameters and local temperature in the lower lip and tongue. **A**: Typical examples of changes in the blood flow (BF, a.u.; arbitrary units), vascular conductance (VC, a.u./mmHg), and local temperature (Tm, °C) in the lower lip (gray traces) and tongue (black traces) on the left side, and SABP evoked by electrical stimulation of the peripheral cut end of the left cervical sympathetic trunk (CST stim.; horizontal bar with dashed lines) for 2 min with a supramaximal intensity (100 µA) at 5 Hz using 2-ms pulses. **B**: Mean ± SEM of decreases in the ∆BF, ∆VC, and ∆Tm in the lower lip (gray symbols) and tongue (black symbols) evoked by cervical sympathetic trunk stimulation at 100 µA and various frequencies (0.5–5 Hz) (n = 6 in each group). Each value is expressed as a percentage of the minimum response. The statistical significance of the differences from base value (at frequency of 0.5 Hz) was assessed by ANOVA followed by a post hoc test (Bonferroni/Dunn test). *P < 0.01, ** P < 0.001 vs. base value (at the frequency of 0.5 Hz). ^†^P < 0.05, ^††^P < 0.01, significant differences between the decreases evoked by cervical sympathetic trunk stimulation in the lower lip and tongue (ANOVA followed by a contrast test). **C**: Typical examples of the changes in blood flow (BF), vascular conductance (VC), and local temperature (Tm) of the lower lip (gray traces) and tongue (black traces) evoked by cervical sympathetic trunk stimulation (2 min, 100 µA, 5 Hz, 2 ms) in combination with lingual nerve stimulation (20 s, 100 µA, 20 Hz, 2 ms). **D**: Mean data ± SEM of the changes in the ∆BF, ∆VC, and ∆Tm of the lower lip (gray bars) and tongue (black bars) evoked by cervical sympathetic trunk stimulation alone (CST), and in combination with lingual nerve stimulation (CST+LN) (n = 6 in each group). *P < 0.05, **P < 0.001 vs. responses evoked by cervical sympathetic trunk stimulation alone at each site (paired *t*-test). ^†^P < 0.001, significant differences between the changes evoked by cervical sympathetic trunk stimulation with lingual nerve stimulation in the lower lip and tongue (unpaired *t*-test). Individual data points are indicated by white markers.

Fig. 5A shows the changes in blood flow, vascular conductance, and local temperature in the lower lip and tongue, as well as the SABP before and after electrical stimulation of the peripheral cut end of the cervical sympathetic trunk. Electrical stimulation for 2 min with 100 µA at 5 Hz (using 2-ms pulses of the left cervical sympathetic trunk) decreased blood flow, vascular conductance, and local temperature in both sites on the left side. Frequency–response curves were generated using stimulus trains (0.5–5 Hz) at 100 µA (Fig. 5B). Significant decreases in the ∆BF, ∆VC, and ∆Tm in the lower lip evoked by cervical sympathetic trunk stimulation occurred above 2 Hz (P < 0.001, ANOVA followed by Bonferroni/Dunn test). Cervical sympathetic trunk stimulation also significantly reduced the ∆BF above 2 Hz and the ∆VC and ∆Tm at 5 Hz in the tongue (P < 0.01, ANOVA followed by Bonferroni/Dunn test).

Decreases in blood flow and vascular conductance in the tongue were significantly larger than those in the lower lip, while the decrease in the local temperature in the tongue was significantly smaller than that in the lower lip when cervical sympathetic trunk simulation was delivered at 5 Hz (ANOVA followed by a contrast test) (Fig. 5B). The HR and SABP remained unchanged during cervical sympathetic trunk stimulation (Table 1). There were no significant differences in the HR and mean SABP before and after cervical sympathetic trunk stimulation (paired *t*-test).

Fig. 5C and D show the effects of cervical sympathetic trunk stimulation (2 min, 100 µA, 5 Hz, 2 ms) in combination with lingual nerve stimulation (20 s, 100 µA, 20 Hz, 2 ms) on blood flow, vascular conductance, and local temperature in the lower lip and tongue. Decreases in blood flow, vascular conductance, and local temperature in both sites evoked by cervical sympathetic trunk stimulation were inhibited by lingual nerve stimulation (Fig. 5C). There were significant differences in the ∆BF (for lower lip, P < 0.05; for tongue, P < 0.001), ∆VC (P < 0.001) and ∆Tm (for lower lip, P < 0.05; for tongue, P < 0.001) in both sites between cervical sympathetic trunk stimulation alone (CST) and in combination with lingual nerve stimulation (CST+LN) (paired *t*-test) (Fig. 5D).

The inhibitory effects evoked by lingual nerve stimulation on the cervical sympathetic trunk stimulation-induced decreases of the blood flow, vascular conductance, and local temperature in the tongue were significantly larger than those in the lower lip (P < 0.001) (unpaired *t*-test). The HR remained unchanged during cervical sympathetic trunk stimulation in combination with lingual nerve stimulation (paired *t*-test) (Table 1). There were significant differences in the SABP before and after cervical sympathetic trunk stimulation in combination with lingual nerve stimulation (P < 0.05) (paired *t*-test).

## Discussion

Our results showed that electrical stimulation of the peripheral portion of the lingual nerve in cervically sympathectomized and vagotomized rats significantly increased blood flow and local temperature in a frequency-dependent manner in the lower lip and tongue on the ipsilateral side (Fig. 2). The observed increases in blood flow and local temperature were mainly the result of parasympathetic vasodilation because (1) vascular conductance at each site was increased significantly by lingual nerve stimulation (Fig. 2), (2) increases in the blood flow and local temperature evoked by lingual nerve stimulation were significantly inhibited by the administration of hexamethonium (Fig. 3), and (3) there were no significant differences in the HR before and after lingual nerve stimulation (Table 1).

The increases in blood flow and local temperature evoked by lingual nerve stimulation were significantly larger in the tongue than in the lower lip (Fig. 2). The findings indicate that parasympathetic vasodilation, driven by trigeminal afferent inputs, is more prominently involved in regulating local temperature in the tongue than in the lower lip. This was supported by findings showing that NADPH diaphorase (demonstrating nitric oxide synthase)-positive and VIP (vasoactive intestinal polypeptide)-like immunoreactive fibers, which are markers of parasympathetic fibers, were most abundant in the lingual, lacrimal, and supraorbital arteries [19].

We were unable to exclude the effects of direct activation of parasympathetic vasodilator fibers derived from facial and/or glossopharyngeal nerves [27], which are involved in the distal part of lingual nerve stimulation on blood flow and local temperature in the orofacial tissues under our experimental conditions. However, the increases in the blood flow and local temperature in the tongue were significantly larger than those in the lower lip during supramaximal stimulation (>10 Hz) of the lingual nerve, which may have induced activation of most nerve fibers in the lingual nerve (Fig. 2). Further, lingual nerve stimulation-induced increases in blood flow and temperature in both sites were prominently inhibited by hexamethonium administration (Fig. 3). These results suggest that parasympathetic activity during trigeminal afferent inputs is critical for regulation of hemodynamics and local temperature in the tongue.

Since the increase in vascular conductance evoked by lingual nerve stimulation in the tongue was not affected by the administration of hexamethonium (Fig. 3), this increase may be due to axon reflex vasodilation mediated by the activation of sensory fibers with collateral branches to the blood vessels of the tongue [28], [29], [30]. Nevertheless, parasympathetic vasodilation appears to be more critical than axon reflex vasodilation in regulating local temperature in the tongue. This is evidenced by the fact that the rise in vascular conductance induced by lingual nerve stimulation during hexamethonium administration did not influence the local temperature of the tongue (Fig. 3).

Increases in blood flow and local temperature evoked by lingual nerve stimulation in the lower lip and tongue were not affected by the intravenous administration of atropine (Fig. 4). This suggests that increases in blood flow and local temperature in the both sites induced by lingual nerve stimulation are mediated mainly by the final neurons via a noncholinergic response. This is in accordance with observations indicating that lingual nerve stimulation-induced blood flow increases in the orofacial skin and oral mucosa are evoked mainly by parasympathetic noncholinergic vasodilator fibers [5], [7], [12], [20], [31].

Although the neural mechanisms underlying noncholinergic parasympathetic vasodilation in the orofacial area are not fully understood, this response may be partly mediated by VIP. Indeed, VIP immunoreactivity is observed in the otic, submandibular, and intralingual ganglion and in nerve fibers innervating blood vessels in the lip [19], [32], [33] and tongue [19], [27], [34], [35]. Furthermore, intravenous administration of VIP induces vasodilation in the lower lip [12], gingiva [7], masseter muscle [36], and submandibular gland [4], which is suppressed markedly by a selective VIP receptor antagonist, as reported previously in the rat.

The mechanism of the increase in SABP induced by lingual nerve stimulation remains unclear. However, trigeminal afferent inputs derived from the periodontal membrane and mechanoreceptors in masticatory muscles were reportedly involved in the pressor response mediated by the efferent pathway of sympathetic nerves using urethane-anesthetized rats [37]. Further, using α-chloralose- and/or urethane-anesthetized rats, stimulation of the nasal mucosa, which is innervated by trigeminal afferent nerves, increased splanchnic sympathetic nerve discharge and elevated arterial blood pressure, and these increases were reduced by injection of a broad-spectrum excitatory amino acid receptor antagonist (kynurenate) into the rostral part of the ventrolateral medulla (RVLM) [38] or injection of CoCl_2_ (a calcium channel blocker) into the medial nucleus of the solitary tract (NTS) [39]. These results suggest that the RVLM and/or NTS may play a role in the brainstem circuit for SABP increase during trigeminal afferent inputs.

Electrical stimulation of the peripheral cut end of the cervical sympathetic trunk significantly decreased blood flow and local temperature in a frequency-dependent manner in the lower lip and tongue on the ipsilateral side (Fig. 5). The decrease induced by cervical sympathetic trunk stimulation appeared to be vasoconstriction because no significant changes in the SABP and HR were observed during the stimulation (Table 1 & Fig. 5). These results indicate that sympathetic vasoconstriction evoked by excess sympathetic activity reduces blood flow and local temperature in orofacial tissues. This is consistent with the previous finding indicating that cervical sympathetic nerve stimulation decreases blood flow and local temperature in the extraoral tissue, such as the lower lip [12]. However, the blood flow decrease with cervical sympathetic trunk stimulation at 5 Hz in the tongue was significantly larger than that in the lower lip, while the cervical sympathetic trunk stimulation-induced local temperature decrease in the tongue was significantly smaller than that in the lower lip (Fig. 5). These results suggest that the two sites differ in the interaction between blood flow and local temperature during sympathoexcitation.

Activation of the cervical sympathetic nerve reportedly induces vasoconstriction in most orofacial tissues mediated by α_1_-adrenoceptors [9], [40]. On the other hand, vasoconstrictions in the tongue surface appear to be mediated primarily by α_2_-adrenoceptors [40]; α_2_-adrenoceptors have been shown to be relatively more prevalent on distal terminal arterioles and inhibited by local metabolic influences, while larger arteriole constriction has been found to be mediated predominantly by α_1_-adrenoceptors and to be less affected by local tissue factors [41]. Therefore, the inhibition of α_2_-adrenergic vasoconstriction evoked by metabolic reactions may be involved in the reduction of hypoperfusion, which induces a decrease in the local temperature of the tongue during sympathoexcitation. Further investigations are necessary to establish the precise mechanisms of the differences in sensitivity to sympathoexcitation between the lower lip and tongue.

Decreases in blood flow and temperature in both the lower lip and tongue evoked by cervical sympathetic trunk stimulation were significantly inhibited by lingual nerve stimulation (Fig. 5). This result indicates that the parasympathetic reflex vasodilation evoked by trigeminal afferent inputs compensates for the hypoperfusion of blood flow, which induces a decrease in local temperature in the orofacial area. Furthermore, the compensatory effect triggered by lingual nerve stimulation in the tongue was notably greater than that observed in the lower lip (Fig. 5). This suggests that, under physiological conditions, the local temperature in the tongue may be maintained at a higher level than that in the lower lip through the activity of the parasympathetic nervous system.

Our findings are consistent with the observation that the local temperature in intraoral tissues is warmer than that in extraoral tissues when the mouth is closed [13]. This elevated temperature is generally considered essential for the proper functioning of intraoral tissues. Indeed, wound repair of the oral mucosa is reportedly faster than that of the skin [42], [43] and the thermosensitive TRPV3 channel contributes to rapid wound healing in oral epithelia [15], [44]. Additionally, the reduced sensitivity to cooling and warmth in the tip of the tongue compared to extraoral tissues, such as the lower lip, may be attributed to the tongue's higher baseline temperature [13].

Components such as saliva or a rich vascular supply may contribute to these intrinsic responses in intraoral tissues [15], [45], [46]. In the present study, similar basal levels of local temperature were recorded in both sites, although the basal levels of blood flow and vascular conductance were significantly higher in the tongue than in the lower lip under resting conditions (Table 2). These observations imply that parasympathetic reflex vasodilation via trigeminal afferent inputs may play a role in the maintenance of local temperature in the intraoral tissues and their functions during mastication, swallowing, and speech, but not during resting oral conditions.

## Conclusion

**Fig. 6:**
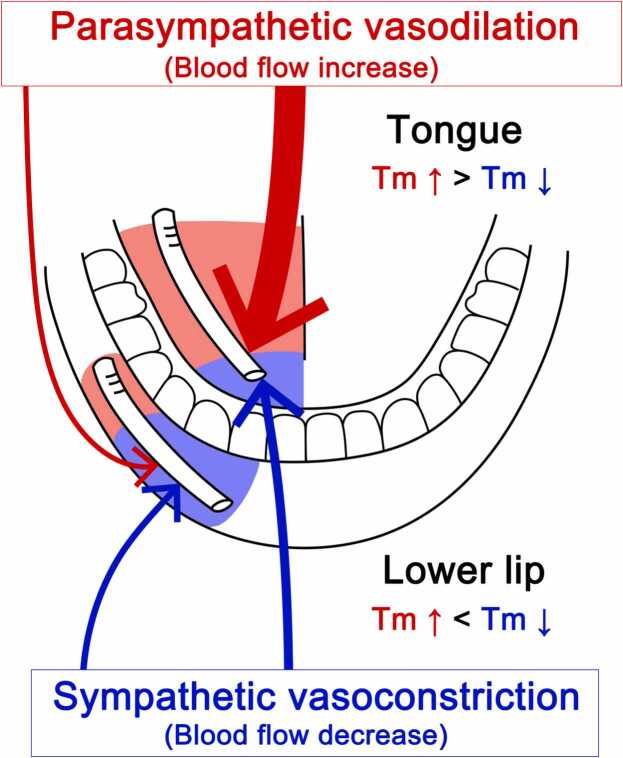
Proposed schema for differences in the regulation of blood flow and local temperature (Tm) in the lower lip (extraoral tissues) and tongue (intraoral tissues) mediated by the autonomic nervous systems. The increases in blood flow and local temperature evoked by parasympathetic vasodilation mediated by trigeminal afferent inputs in the tongue were significantly greater than those in the lower lip. Sympathetic vasoconstriction evoked by excess sympathetic activity reduced blood flow and local temperature in both tissues. These decreases were restored by parasympathetic reflex vasodilation; the effects were larger in the tongue than in the lower lip. Therefore, the local temperature in the tongue may be maintained at a higher level than that in the lower lip through the activity of the parasympathetic nervous system.

In conclusion, our results suggest that parasympathetic vasodilation evoked by trigeminal afferent inputs is involved in the maintenance of hemodynamics and local temperature in the orofacial area, and the effects may be greater in the tongue (intraoral tissues) than in the lower lip (extraoral tissues). Parasympathetic reflex vasodilation increases blood flow and local temperature, which may help compensate for hypoperfusion and the decrease in local temperature mediated by the cervical sympathetic trunk (Fig. 6).

Further investigation into the precise neural mechanisms and molecular interactions governing the relationship between hemodynamics and local temperature in orofacial tissues during trigeminal afferent inputs will enhance our understanding of the autonomic nervous system's role in orofacial functions, including oral sensation and wound healing. In addition, continued research could shed light on the etiology of orofacial disorders, such as burning mouth syndrome (glossalgia) and fibromyalgia, which currently lack definitive clinical findings.

## CRediT authorship contribution statement

**Toshiya Sato:** Writing – review & editing, Investigation. **Hanako Ohke:** Writing – review & editing, Investigation. **Mari Shimatani:** Writing – review & editing, Investigation. **Hisayoshi Ishii:** Writing – review & editing, Writing – original draft, Investigation, Funding acquisition, Data curation, Conceptualization. **Syed Taufiqul Islam:** Writing – review & editing, Writing – original draft, Investigation, Data curation.

## Authors' contributions

STI, HI make conception and design of research. STI, HI, TS, HO, MS performed experiments. STI, HI analyzed data. STI, HI interpreted results of experiments. STI, HI prepared figures. STI, HI drafted manuscript. All authors edited and revised manuscript. All authors read and approved the final manuscript.

## Consent for publication

All authors fully agree with the submission and publication of this manuscript in this journal.

## Ethics approval and consent to participate

The experimental protocols were reviewed and approved by the Animal Ethics and Research Committee and conducted in accordance with the Regulations for the Care and Use of Laboratory Animals of the Health Sciences University of Hokkaido (No. 24–038). All the animals were cared for in accordance with the recommendations in the current National Research Council guide.

## Funding

This study was partly supported by MEXT KAKENHI (No. 23K09280; to H. Ishii).

## Declaration of Competing Interest

The authors declare that they have no known competing financial interests or personal relationships that could have appeared to influence the work reported in this paper.

## Data Availability

All relevant data are within the paper.
